# Formation and Stability of Low‐Dimensional Structures for Group VIIIB and IB Transition Metals: The Role of sd^4^ Hybridization

**DOI:** 10.1002/advs.201500314

**Published:** 2016-01-21

**Authors:** Jianhui Yang, Qiuju Zhang, Liang Chen, Gang Wang, Xiaolong Chen

**Affiliations:** ^1^ Ningbo Institute of Materials Technology and EngineeringChinese Academy of SciencesNingboZhejiang315201P.R. China; ^2^ Research and Development Center for Functional CrystalsBeijing National Laboratory for Condensed Matter PhysicsInstitute of PhysicsChinese Academy of SciencesBeijing100190P.R. China; ^3^ Collaborative Innovation Center of Quantum MatterBeijing100190P.R. China

**Keywords:** Au nanowires, face‐centered cubic, first principles, sd hybridization, transition metal nanostructures

## Abstract

**A quasi‐sd^4^ hybridization state for group VIIIB and IB face‐centered cubic (FCC) transition metals in low‐dimensional nanostructures** is identified, in contrast to the sd^5^ hybridization state in bulk. For Au, a novel three‐shelled nanowire is designed with a hexagonal close‐packed core in the sd^5^ hybridization, wrapped by FCC‐(111) shell that adopts the quasi‐sd^4^ hybridization. This new nanostructure exhibits remarkable stability and electronic properties.

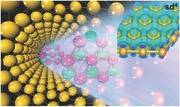

Since the discovery of beautiful low‐dimensional carbon nanostructures, such as fullerene,^1^ carbon nanotubes and graphene,^2^, ^3^ tremendous efforts have been devoted to exploring similar nanostructures for transition metals (TMs) and exploiting their potential applications in electronic devices and selective catalysis.^4^, ^5^, ^6^, ^7^, ^8^, ^9^, ^10^ To date, various carbon‐like low‐dimensional TM, particularly in Group VIIIB and IB, nanostructures have been successfully realized. For example, Huang et al. proved the existence of Au nanocages with similar structures as fullerenes as shown by photoelectron absorption experiments and first principles calculations.^11^ For the 1D case, Ugarte et al. observed the formation of the smallest Ag nanotube with a square cross‐section during the elongation of Ag nanocontacts.^12^, ^13^ Kondo et al. prepared helical Pt and Au nanowires and nanotubes with an electron beam thinning technique.^14^, ^15^ These 1D TM small size nanostructures preferentially adopt hollow or multiwalled structures, which are exactly the analogue of carbon nanotubes. For the 2D case, Li et al. successfully fabricated organic ligand‐supported Rh nanosheets by a facile solvothermal method.^4^ Another example is the template‐synthesized folding Pd_11_ nanosheet through the treatment of a ladder polysilane.^5^ In both examples, the planar single‐layer structures were stabilized by the TM–ligand interactions.

Among the TMs, Au has received particularly intensive attention. A variety of stable Au nanocages, nanowires, and nanotubes have been successfully fabricated.^6^, ^14^, ^16^, ^17^, ^18^ Correspondingly, many mechanistic studies have been conducted and unraveled the structural evolution of small Au nanoclusters with size. Essentially, these studies attempted to ascertain the difference of Au from other TMs (i.e., the uniqueness of Au usually associated with the scalar‐relativistic effect) and explained why Au can form these carbon‐like structures.^19^, ^20^, ^21^, ^22^ However, as mentioned above, many other TMs can also form the carbon‐like structures, but with lower stability. We believe that there should exist some generic mechanism for the formation and stability of TM low‐dimensional nanostructures, which is still far unclear.

Indeed, the fundamental mechanism is critical for understanding the properties and exploring the applications of TM nanostructures. In particular, the surface electron state, which directly determines the physical and chemical properties of TM nanostructures, would be easily unraveled on the basis of the formation mechanism. In the present study, we revealed the dimensionality‐driven hybridization of s and d electron orbitals in the face‐centered cubic (FCC) TMs including six group VIIIB (Co, Ni, Rh, Pd, Ir, and Pt) and three group IB (Cu, Ag, and Au) elements. Note that Co can exist in both FCC and hexagonal close‐packed (HCP) phases, and the energy difference between them is smaller than 0.03 eV atom^−1^.^23^ Various 0D, 1D, and 2D Au nanostructures were considered and compared in this study (see Figure S1, Supporting Information). A new quasi‐sd^4^ hybridization state involving s‐orbital and four d orbitals in a low dimension was identified, in contrast to the sd^5^ hybridization state involving all s and d orbitals in bulk. We proposed a control parameter to evaluate the stability of the quasi‐sd^4^ hybridization state for all FCC metals, and the stability of their low‐dimensional nanostructures. Au low‐dimensional nanostructures are found to possess the highest stability due to the number of valence electrons and scalar‐relativistic effect. Using the 1D Au nanostructures as an example, we demonstrated how the hybridization states determine the structure—particularly the low‐dimensional nanostructures and physicochemical properties.

Let us start from the analysis on Au, which is the most representative TM with carbon‐like nanocages, nanowires, and nanotubes. We have investigated over 70 different low‐dimensional Au nanostructures (see Figure S1, Supporting Information). Apparently, the similarity of Au and carbon nanostructures implies that the electronic structures of both elements share some similar features. For carbon, the major difference in electronic structures for 2D (graphene) and 3D (diamond) structures is whether the 2p_z_ orbitals are hybridized with 2s, 2p_x_, and 2p_y_ orbitals (i.e., yielding the sp^2^ and sp^3^ hybridization states, respectively). In light of this distinction, we also compared the electronic structures of 2D layer and 3D FCC bulk for Au. It is known that the 5d and 6s orbitals are hybridized when Au–Au bonds are formed. As shown in **Figure** **1**, all 5d and 6s orbitals are resonant across a wide energy range (−7 to 0 eV) in the perfect FCC Au structure. In contrast, the d_z_2 orbitals in the perfect 2D structure (i.e., a single Au(111) layer); termed as L_1_ hereafter, are quite localized across a narrow energy range (−3.5 to −1.7 eV) and have smaller energy resonant ranges with 6s and other 5d orbitals. Moreover, electrons assemble into the d_z_2 orbital from the *xy*‐plane, as shown in the right panel of Figure 1. Here, the d_z_2 orbitals refer to the orbitals along the surface normal. This suggests that d_z_2 orbitals have a rather small contribution to the Au–Au bond in L_1_. Herein, we refer this type of hybridization state in L_1_ to quasi‐sd^4^, and refer the hybridization state in perfect 3D FCC structure to sd^5^. For the quasi‐sd^4^ hybridization, the d_z_2 orbital is quite localized with narrow energy range and the d_xz_ and d_yz_ orbitals involving the z‐direction components are also localized with slightly wider energy range. For the sd^5^ hybridization, d_z_2, d_xz_, and d_yz_ orbitals have similar energy resonance range and shape. Apparently, in an Au entity, only the under‐coordinated surface atoms can adopt the quasi‐sd^4^ hybridization state, while the saturated inner atoms still prefer sd^5^. Thus, L_1_ and the derived structures (e.g., hollow cages and nanotubes) are extreme cases for the quasi‐sd^4^ hybridization state because all atoms are under‐coordinated surface atoms.

**Figure 1:**
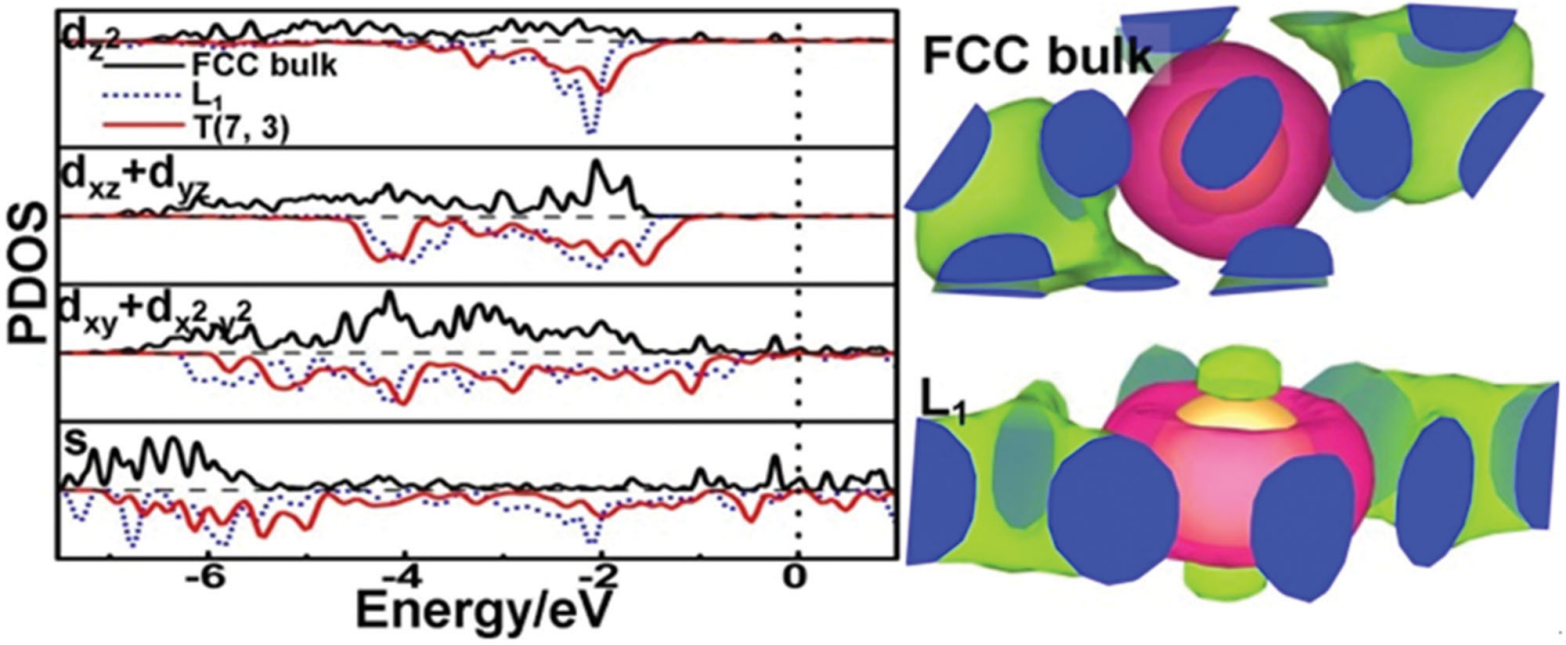
(Left panel): PDOS of L_1_, bulk Au, and T(7,3). Fermi level was shifted to zero. (Right panel): Charge difference of FCC bulk Au and L_1_. The green and red colors stand for gain and loss of electrons in the formation of Au–Au bonds, respectively.

Perfect quasi‐sd^4^ hybridization only exists in the case of L_1_. Bending the sheet or introducing extra Au–Au bonds would force the d_z_2 orbitals to participate in hybridization and destroy the perfect quasi‐sd^4^ hybridization.^24^ To illustrate such variations of d_z_2 orbitals, projected density of states (PDOS) of L_1_ and Au nanotube T(7,3) with a curved surface is displayed in Figure 1. For L_1_, the d_z_2 orbitals are fully filled and located far away from the Fermi level. The d_z_2 orbitals are weakly polarized as L_1_ is rolled into T(7,3) with some higher energy antibonding states in the range of −2 to −1.5 eV. In the meantime, the energy of d_xy_ and d_x_2_‐y_2 orbitals in the *xy*‐plane shifts up, and the density of low energy states between −7 and −6 eV decreases. Overall, the quasi‐sd^4^ hybridization is weakened, and cohesive energy (*E*_c_) is decreased. Therefore, *E*_c_ of L_1_ (2.88 eV atom^−1^) is the upper bound for these hollow 0D cages, 1D tubes, and 2D layer—all of which are derived from the planar L_1_ sheet. As shown in Figure S2 of the Supporting Information, the *E*_c_ of cages are generally smaller than that of tubes with a similar radius because 0D cage structures are more distorted than 1D tube structures.

In fact, similar hybridization states also exist in other eight TMs (Figure S3, Supporting Information). To evaluate the stability of quasi‐sd^4^ hybridization states for different TMs, we defined a control parameter *R*_C_ that is the ratio of *E*_c_ for L_1_ to FCC bulk. Clearly, higher *R*_C_ value indicates higher stability of the quasi‐sd^4^ hybridization state and thus higher stability of the associated low‐dimensional structures. **Figure** **2** shows that this parameter correlates to the number of valence electrons (*N*_ve_), which increases when *N*_ve_ increases from 9 to 11. In principle, the *E*_c_ of the bulk decreases as *N*_ve_ increases due to the fewer unfilled orbitals. In this study, we show that the *E*_c_ of L_1_ also obeys this rule. As shown in Figure 1, the main difference between the PDOS of L_1_ and FCC bulk is the localization of d_z_2 states. The average energy of the d_z_2 states is higher than that of s and other four d states. As a result, L_1_ has lower *E*_c_ than the FCC bulk, which leads to *R*_C_ values below 1. As the *N*_ve_ increases, the nucleus holds electrons more firmly. The tendency to fill electrons in the low‐energy s and d states would be stronger in L_1_. In turn, *R*_C_ increases.

**Figure 2:**
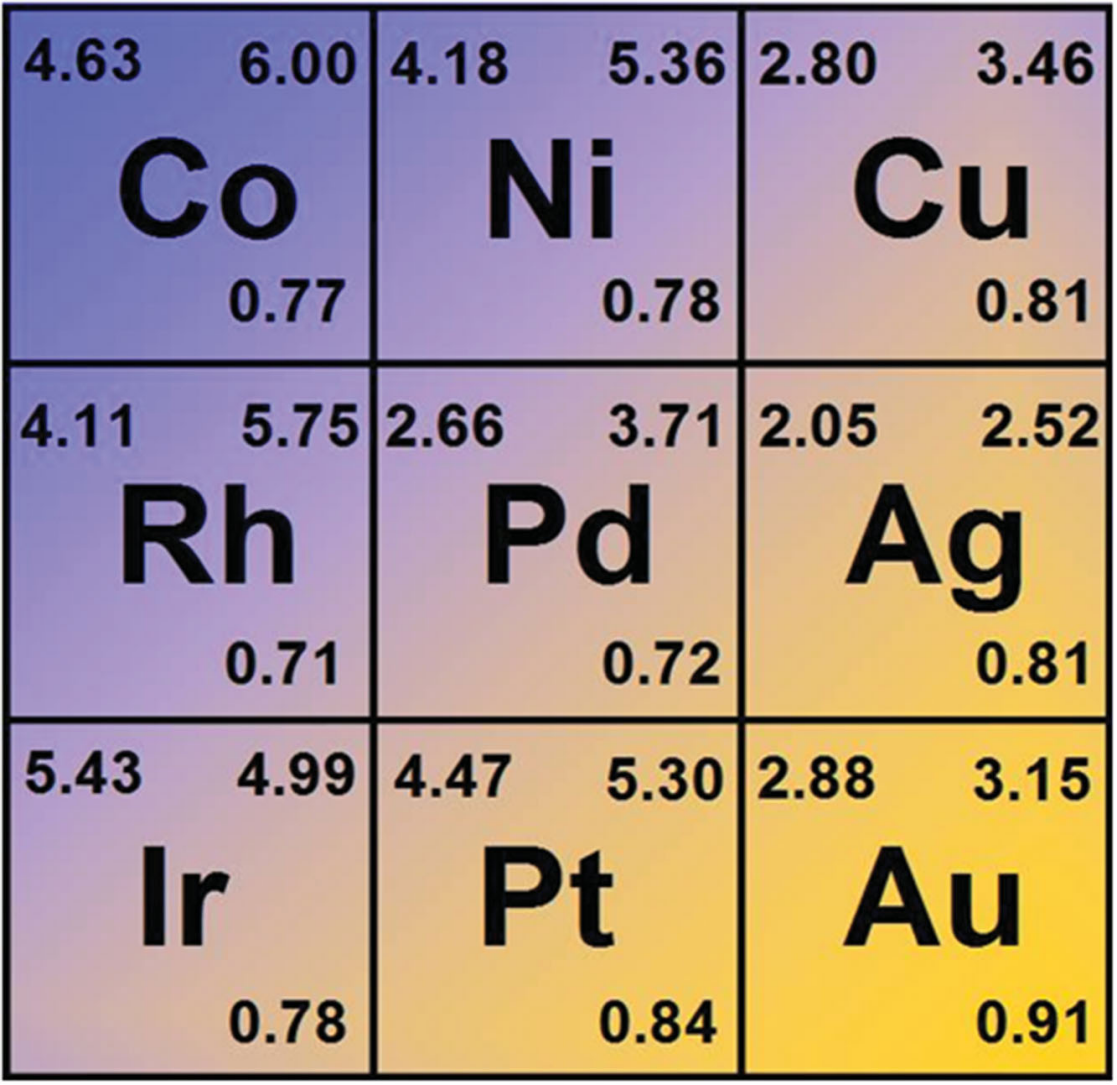
The calculated *E*_c_ (unit: eV) of L_1_ (upper left), FCC bulk (upper right), and *R*_C_ (lower right) for different TMs.

On the other hand, for the TMs of same group, 5d TMs generally have a higher *R*_C_ than 3d and 4d TMs due to the scalar‐relativistic effect. If the scalar‐relativistic effect was excluded, the *R*_C_ of the TMs of same group would be very close (Figure S4, Supporting Information). Particularly, Cu, Ag, and Au have virtually identical *R*_C_ in the nonscalar‐relativistic calculations. The contraction of s orbitals caused by the scalar‐relativistic effect shortens the bond lengths in both the L_1_ and FCC bulk (Figure S5, Supporting Information). This enhances *E*_c_.^19^ The bond lengths in L_1_ are shortened more significantly than FCC bulk. However, the contraction of s orbitals also increases the shielding effect of d electrons and increases the energy level. As a result, the d orbital expanding reduces *E*_c_. In L_1_, the d_z_2 orbitals are mainly distributed along the *z*‐axis and far away from s orbitals (mainly in *xy*‐plane). This reduces the shielding effect between the s and d orbitals. Therefore, the increased energy level (i.e., weakened *E*_c_) caused by the scalar‐relativistic effect is less significant for L_1_ than bulk. Accordingly, a higher *R*_C_ is seen for 5d TMs.

Overall, both factors (*N*_ve_ and scalar‐relativistic effect) give rise to the highest *R*_C_ for Au among the studied TMs. Indeed, Au prefers planar structures more than Pt and Ag, the critical number for 2D‐to‐3D transition (i.e., from planar belts to clusters) being 7,^19^ 10,^25^ and 14^26^ for Ag, Pt, and Au, respectively. This is quite consistent with the order of *R*_C_. Note that the planar belt is cut from L_1_ along one specific direction and has dangling bonds on the edges. Moreover, for Au itself, anionic nanoclusters with extra valence electrons have more preferable 2D planar structures than cationic nanoclusters.^27^

Now let us look again at carbon. Carbon has a large *R*_C_ (>1) because the sp^2^ carbon structures are more stable than the sp^3^ carbon structures in terms of thermodynamics. However, both of them can stably exist because of the high energy barrier for the sp^2^–sp^3^ hybridization transition. Unlike carbon, Au and other TMs have lower *R*_C_ values (less than 1). There is no energy barrier for the quais‐sd^4^ to sd^5^ transition. As a result, the above quasi‐sd^4^ hybridized Au structures are not stable enough and thus they can only exist in small scales or low‐dimensions in which the under‐coordinated surface atoms are dominant. At some threshold size, they will spontaneously transform to the sd^5^ hybridized structures. Indeed, the experimentally prepared TM nanosheets must be stabilized by protecting ligands to avoid converging to sd^5^ hybridized structures.^3^, ^4^ This implies that some size‐dependant structural evolution may occur.

We are aware that many theoretical and experimental studies have already been conducted for Au nanoclusters and concluded that Au nanoclusters undergo evolution with respect to size: the most stable structure of Au_n_ clusters is planar for n < 14,^22^ cage for 16 ≦ n ≦ 20,^11^, ^16^ and filled structures for n > 20.^21^ In the present work, we placed our attention on the 1D Au nanostructures. We defined the atomic linear density (ρ_L_), which is the number of Au atoms in a unit length to represent the “size” of 1D structures. Interestingly, we found that the evolution of 1D Au nanostructures with size have similar behavior to the nanoclusters. As shown in **Figure** **3**, planar belts are the most stable structures for ρ_L_ < 2.41 Å^−1^ [corresponding to ρ_L_ of T(6,1)], while hollow tubes become the most stable for 2.41 Å^−1^ < ρ_L_ < 4.43 Å^−1^ [corresponding to ρ_L_ of T(12,6)].

**Figure 3:**
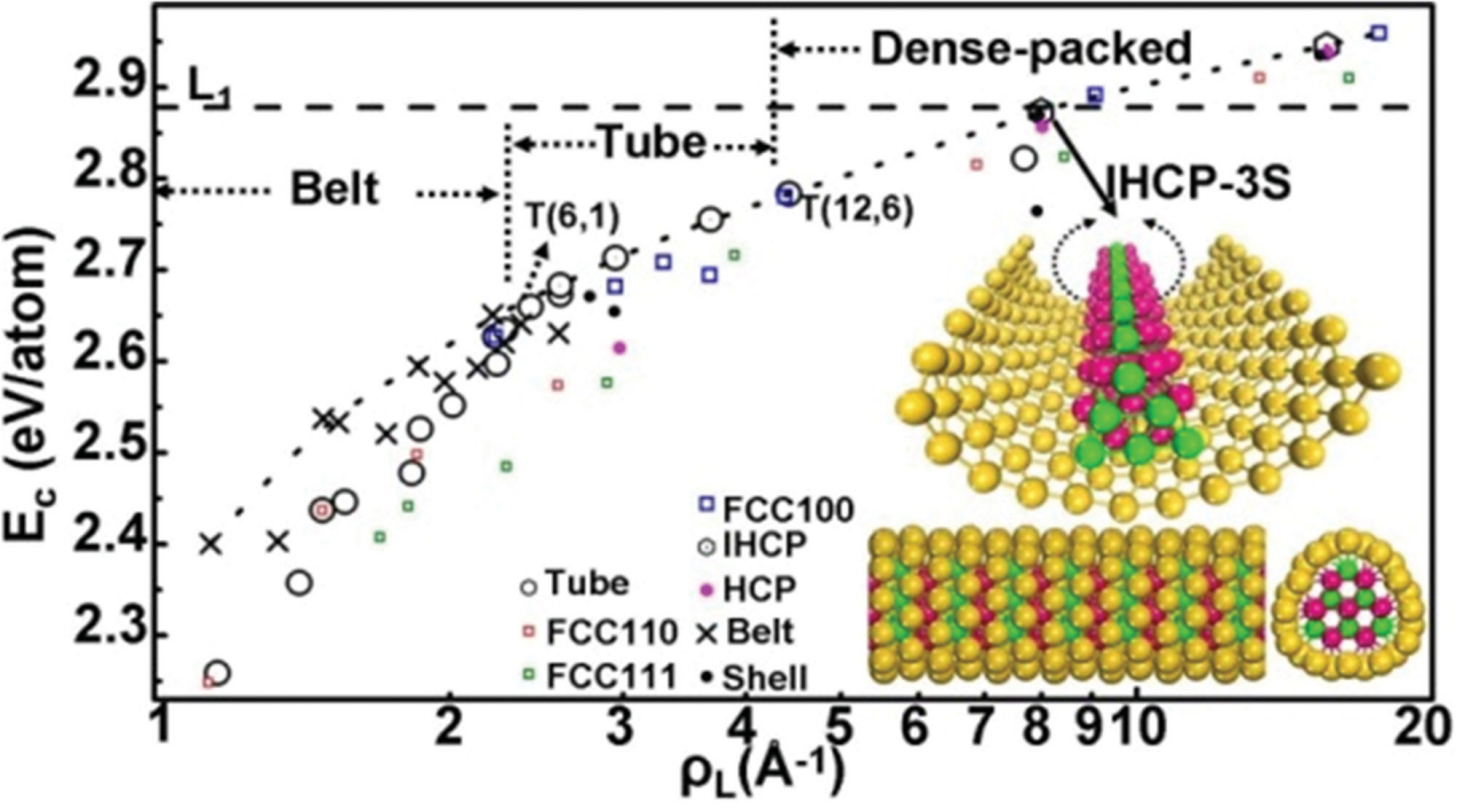
The structural evolution of 1D Au nanostructures. The structures of each Au entities are shown in Figure S1 of the Supporting Information.

Compared to L_1_, the belts have lower *E*_c_ because of dangling bonds on the edge. Rolling belts into tubes can eliminate the dangling bonds, while the curvature would incur energy penalty and decreases *E*_c_. Thus, planar belt structures are preferred for small ρ_L_. On the contrary, tube structures are preferred for large ρ_L_ because the energy penalty from curvature is relatively small. Actually, the same trend is also applicable for the variation of 0D structures from a planar island to cages. When ρ_L_ is larger than 4.43 Å^−1^, the filled structures with inner Au atoms become the most stable because the inner atoms are usually saturated, and their valence electrons adopt the more stable sd^5^ hybridization. Correspondingly, the *E*_c_ of these filled structures exceed the upper bound for hollow tube structures.

For a TM structure, the inner atoms prefer the most stable and close‐packed crystalline structures, while surface atoms adopt the second stable quasi‐sd^4^ hybridization and prefer the smooth and densest‐packed arrangement. This has the lowest surface energy. The different and competitive tendencies of surface and inner atoms determine the final structures.^28^ Apparently, if the number of surface atoms is dominant, the most stable inner close‐packed structures would be sacrificed. As a result, spherical nanoclusters and cylindrical nanowires would be formed in the 0D and 1D cases, respectively. This is confirmed by geometric optimization of the nanowires truncated from bulk FCC TMs. We found that these truncated nanowires have smaller *E*_c_ than belts and tubes in a small ρ_L_. In particular, FCC110–4R, FCC110–6R, and FCC100–3R (*n*R represents the number of atomic rows) are not stable and would transform to hollow tubes upon structural optimization as shown in Figure S6 of the Supporting Information. For example, the (100) face of FCC100–3R is reconstructed to the (111) facet accompanied with a disappearance of the inner Au–Au bonds. Correspondingly, the original FCC100–3R structure would eventually evolve to a hollow T(6,3) nanotube. In fact, these FCC100 Au nanowires are unstable due to their high surface activity, while FCC110 nanowires are much more stable.

On the contrary, the inner close‐packed structures are preferred, and the smooth surface is destroyed if the number of surface atoms is sufficiently small. Experimentally, the HCP phase has been observed in Au nanoparticles.^29^ As such, we designed a new three‐shelled Au 1D nanostructure, which has a HCP interior core wrapped by a curved FCC‐(111) shell. Note that the HCP arrangement can form a high‐symmetry 1D structures and can be wrapped by a smooth surface—the FCC arrangement cannot. Hereafter, we will refer to this structure as shown in the inset of Figure 3 as IHCP‐3s. Interestingly, we found that IHCP‐3s is the most stable one among the structures with similar ρ_L_ values (see Figure S7, Supporting Information), even including the experimentally synthesized nanowires.^14^ Therefore, we expect that such structures can be experimentally prepared. Some recent experimental progresses are supportive and inspiring. For example, Kondo and Takayanagi have prepared the helical Au nanowires by electron beam thinning technique.^14^ Ugarte and co‐workers obtained Ag nanotubes during the elongation of Ag nanocontacts.^13^ Considering the higher energetics than that of these metastable structures, we propose that the IHCP‐3s Au structure can be achieved by thinning Au wires down to a diameter of around 1.5 nm in vacuum or noble gas protection, followed by a thermal annealing. The vacuum or noble gas protection is necessary because the adsorption of other molecules may induce undesired surface reconstruction. Furthermore, it should be pointed out that the stability of the IHCP‐3s Au structures is closely determined by the stability of quasi‐sd^4^ state and the resulting *R*_C_ values. For example, Ag and Cu do not have stable IHCP‐3s structures due to their smaller *R*_C_.

In addition to the thermal stability, we next evaluated the surface stability of IHCP‐3s versus FCC110–3s′, Au_38_, and Au (111) surfaces using the adsorption of various chemicals as a probe. The calculations (see **Figure** **4**) indicate that the order of surface activity is as follows: Au_38_ > FCC110–3s′ > IHCP‐3s ≈ Au (111). The surface activity of IHCP‐3s is quite low and comparable to that of Au(111). Particularly, O_2_ and C_2_H_4_ can only be physisorbed on IHCP‐3s, but can be chemisorbed on Au_38_ and FCC110–3s′. Furthermore, the dissociation barrier of O_2_ on IHCP‐3s is calculated to be 1.09 eV, suggesting a low probability of dissociation at ambient conditions.

**Figure 4:**
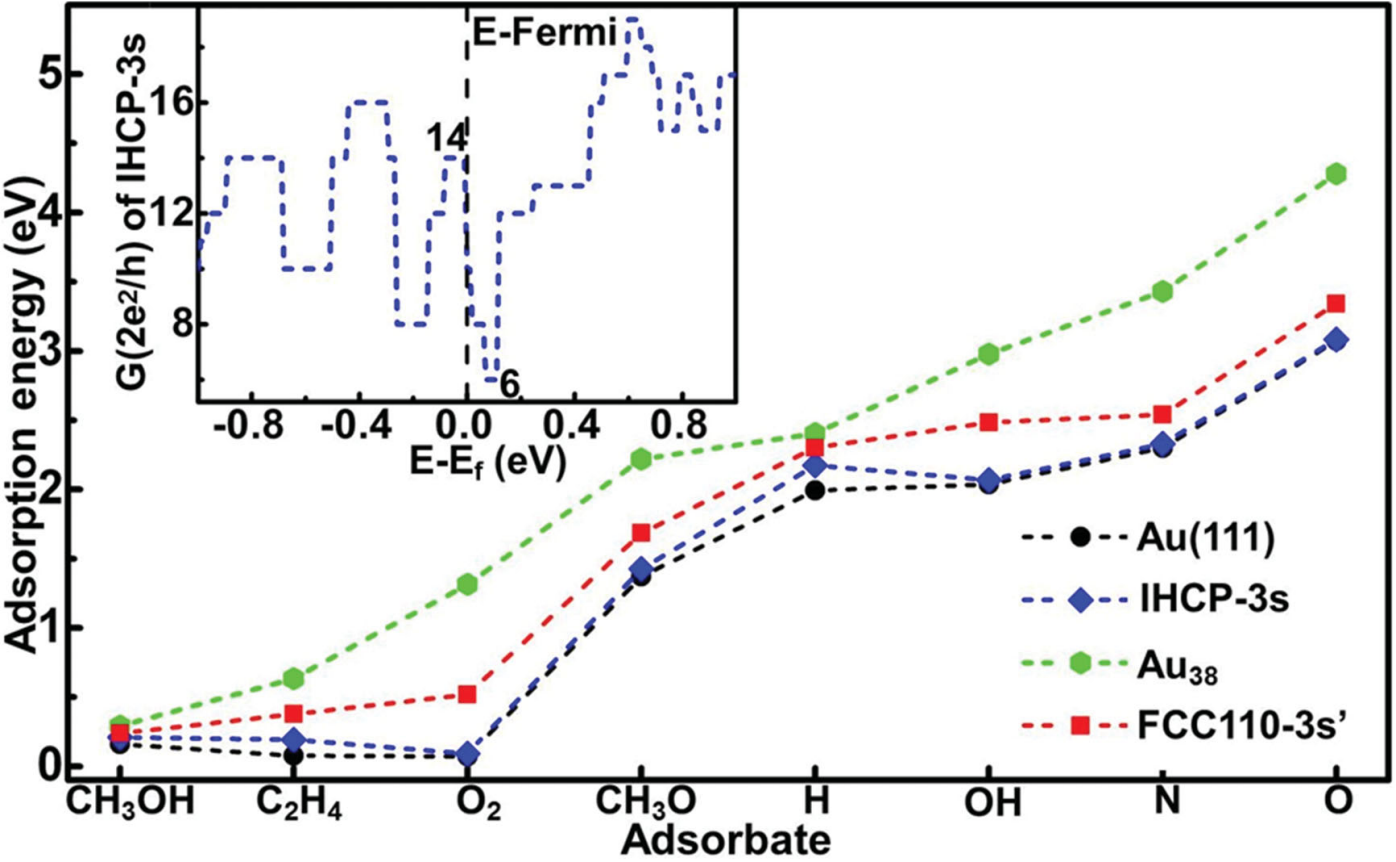
The comparison of surface activity of different Au entities. The inset displays the calculated conductance of IHCP‐3s near the Fermi level.

The intrinsic origin of the low surface activity of IHCP‐3s can be explained by the PDOS of inner (Au^I^) and surface atoms (Au^S^). As shown in Figure S9 of the Supporting Information, the d_z_2 orbitals of Au^S^ is similar to that of L_1_, which is mainly localized in the energy range from –4 to –1 eV and far away from the Fermi level. It indicates that the d_z_2 orbitals of Au^S^ are nearly filled. According to Hammer and Norskov's theory, Au is the most inert TM partly because of its high filling degree of d bands.^30^ In the case of 1D Au nanostructures, the d_z_2 orbital is the major component of d bands to participate in the reaction and interaction with upcoming chemical molecules. Thus, the nearly filled feature of d_z_2 orbitals leads to the low surface activity of IHCP‐3s. On the other hand, Au^I^ and Au atoms in bulk share a similar feature in PDOS. Clearly, the low surface adsorption activity implies that IHCP‐3s may be chemically stable after being exposed to air. This is very advantageous because applications of nanostructures are usually hindered by their high surface activity particularly when subjected to oxidation in air.

It is known that nanostructures usually have an extreme curvature on their edges, which leads to low work function due to the point effect. In contrast, IHCP‐3s has a high work function (5.33 eV), which stems from the smooth surface that constitutes the quasi‐sd^4^ hybridized atoms. This value is similar to that of Au(111) (5.31 eV).^31^ The high work function of IHCP‐3s makes electrons hard to escape from the surface, which may ensure the stability of current in electronic devices. Finally, we calculated the conductance of IHCP‐3s to investigate the electron transport properties. As shown in the inset of Figure 4, IHCP‐3s shows notable quantum conductance around the Fermi level. The conductance of IHCP‐3s is 14 G_0_ below the Fermi level (≈ −0.01 to −0.1 eV). It decreases to 6 G_0_ in the range of 0.02–0.11 eV. This means that the conductance can be tuned more than twofold by changing the gate bias. For comparison, the FCC110—3s′ with similar radius—‐which is the most stable truncated nanowires in the FCC arrangement—has weaker quantum conductance variations (15 to 18 G_0_) around the Fermi level. Combined with the high chemical and thermal stability, we anticipate that IHCP‐3s may be a promising material for nanoelectronics. Indeed, we also found that a four‐shelled IHCP‐4s structure is energetically favorable and exhibits similar features with IHCP‐3s. However, this feature would disappear when the IHCP wire becomes thicker. This is consistent with the aforementioned structural evolution.

In summary, we identified a relatively stable quasi‐sd^4^ hybridization state in addition to the sd^5^ hybridization state for FCC TMs. The stability of quasi‐sd^4^ hybridization state is determined by the number of valence electrons and scalar‐relativistic effect. Both factors enable Au to adopt the stable nanostructures with quasi‐sd^4^ hybridized states. For Au nanostructures, the surface atoms prefer smooth FCC(111)‐like surface in quasi‐sd^4^ hybridization, while inner atoms prefer close‐packed arrangements in sd^5^ hybridization. Competition of these two tendencies determines the final structural arrangements of Au nanostructures. We designed a stable three‐shelled IHCP structure, which could be a promising material for electronic devices owing to the high stability and large quantum conductance variations around the Fermi level.

Spin‐polarized density functional theory (DFT) computations were performed using the Vienna ab initio simulation package (VASP).^32^ The PW91 exchange‐correlation functional^33^ was employed with the electron–ion interactions described by the projector augmented wave (PAW) potentials.^34^ We have also comparatively applied PBE functional and found essentially consistent results. The scalar‐relativistic effect and spin–orbit coupling were included for 5d TMs. Full structural optimizations were performed until the force on each atom was less than 0.02 eV Å^−1^. The density of K‐points in real space was less than 0.034 × 0.034 × 0.034 Å^−3^ for all calculations, based on the Monkhorst–Pack method.^35^ The transition states for reactions were identified by using the climbing nudged elastic band method (cNEB)^36^ and further confirmed by the frequency calculations. *E*_c_ used to describe the stability of these systems was calculated as following: *E*_c_ = *E*_M_ – *E*_t_/*N*_t_, where *E*_M_ is the energy of single atom, *E*_t_ is the total energy of systems, and *N*_t_ is the total number of atoms.

This is an open access article under the terms of the Creative Commons Attribution License, which permits use, distribution and reproduction in any medium, provided the original work is properly cited.

## Supporting information

As a service to our authors and readers, this journal provides supporting information supplied by the authors. Such materials are peer reviewed and may be re‐organized for online delivery, but are not copy‐edited or typeset. Technical support issues arising from supporting information (other than missing files) should be addressed to the authors.

Supplementary
