# Supplementary material for: Formation and Stability of Low‐Dimensional Structures for Group VIIIB and IB Transition Metals: The Role of sd4 Hybridization
Source: Adv Sci (Weinh). 2016 Jan 21;3(4):1500314. doi: 10.1002/advs.201500314 (PMC5115452; doi:10.1002/advs.201500314)
Supplement: Supplementary file 1 — Supplementary [file ADVS-3-0i-s001.pdf]

Copyright WILEY-VCH Verlag GmbH & Co. KGaA, 69469 Weinheim, Germany, 2016.

ADVANCED SCIENCE
Open Access

Supporting Information

for *Adv. Sci.*, DOI: 10.1002/advs. 201500314

Formation and Stability of Low-Dimensional Structures for Group VIIIB and IB Transition Metals: The Role of sd^4 Hybridization

Jianhui Yang, Qiuju Zhang, Liang Chen, Gang Wang,* and Xiaolong Chen**

DOI: 10.1002/((please add manuscript number))
Article type: Communication

Formation and Stability of Low-dimensional Structures for Group VIIIB and IB Transition Metals: the Role of sd^4 Hybridization

Jianhui Yang, Qiuju Zhang, Liang Chen, Gang Wang* and Xiaolong Chen**

Dr. J.H. Yang, Dr. Q.J. Zhang, Prof. L. Chen
Ningbo Institute of Materials Technology and Engineering
Chinese Academy of Sciences,
Ningbo, Zhejiang 315201, China
E-mail: chenliang@nimte.ac.cn
Prof. G. Wang
Research & Development Center for Functional Crystals
Beijing National Laboratory for Condensed Matter Physics, Institute of Physics, Chinese Academy of Sciences, Beijing 100190, China
E-mail: gangwang@iphy.ac.cn
Prof. X.L. Chen
Research & Development Center for Functional Crystals
Beijing National Laboratory for Condensed Matter Physics, Institute of Physics, Chinese Academy of Sciences, Beijing 100190, China
Collaborative Innovation Center of Quantum Matter, Beijing, China
E-mail: chenx29@iphy.ac.cn

Keywords: sd hybridization, face-centered cubic, transition metal nanostructures, first principles, Au nanowires

Copyright WILEY-VCH Verlag GmbH & Co. KGaA, 69469 Weinheim, Germany, 2013.

Supporting Information

Formation and Stability of Low-dimensional Structures for Group VIIIB and IB Transition Metals: the Role of sd^4 Hybridization

Jianhui Yang, Qiuju Zhang, Liang Chen, Gang Wang* and Xiaolong Chen**

Figure S1. Various gold structures investigated in this study

1.1. The structures and E_c (unit = eV) of Au_n clusters

a. Planar and cage-like structures of Au_4

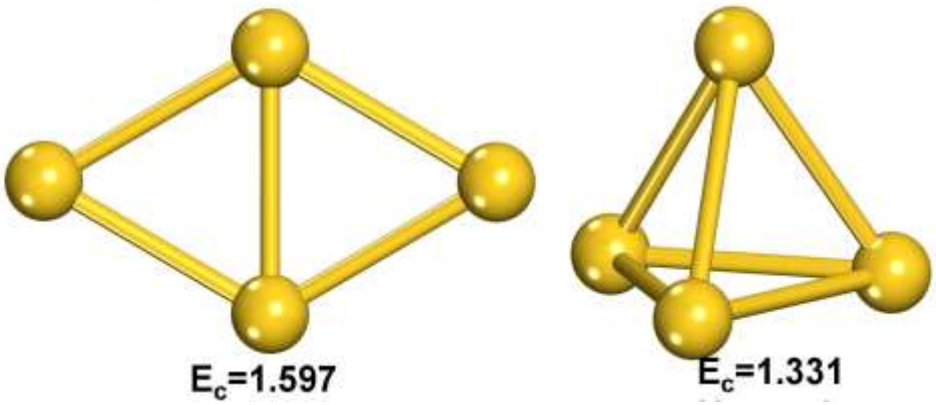

b. Planar and cage-like structures of Au_6

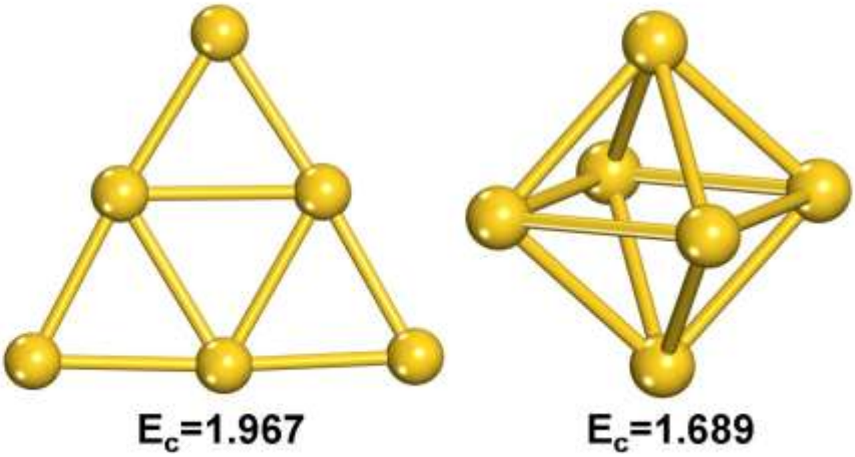

c. Planar and cage-like structures of Au_{12} and filled structure of Au_{13}.

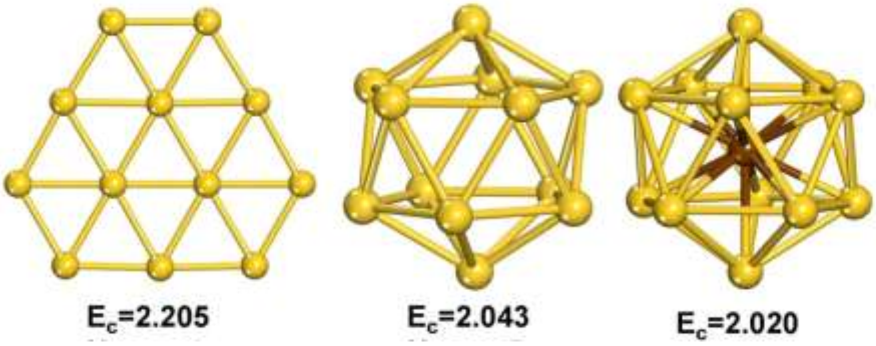

d. Planar and cage-like structures of Au_{16}

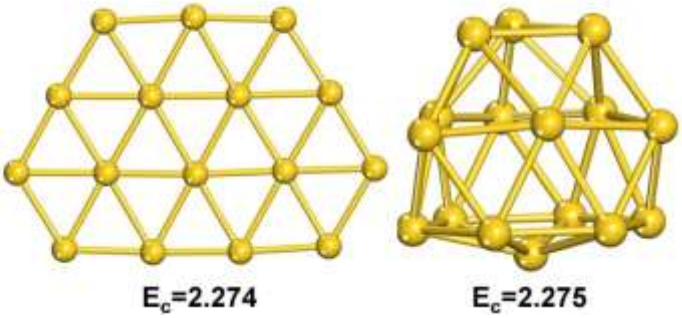

e. Cage-like structures of Au_{42} and Au_{92}

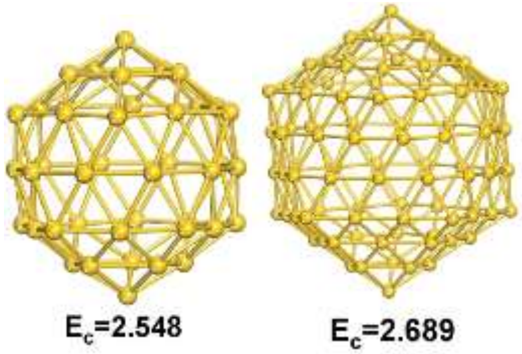

1.2. One-dimensional (1D) Structures

For the notation 'FCC110-nR', nR means the number of atomic rows. FCC110 and FCC111 represent the (110) and (111) facet layer that is rolled to form tube structures, respectively. T(n,m) is the chiral nanotube, in which n and m are the two chiral indices. The belts are cut from L_1, and periodic along x direction with dangling bonds on the boundary.

a. The side-view of 1D Au structures with three atomic rows:

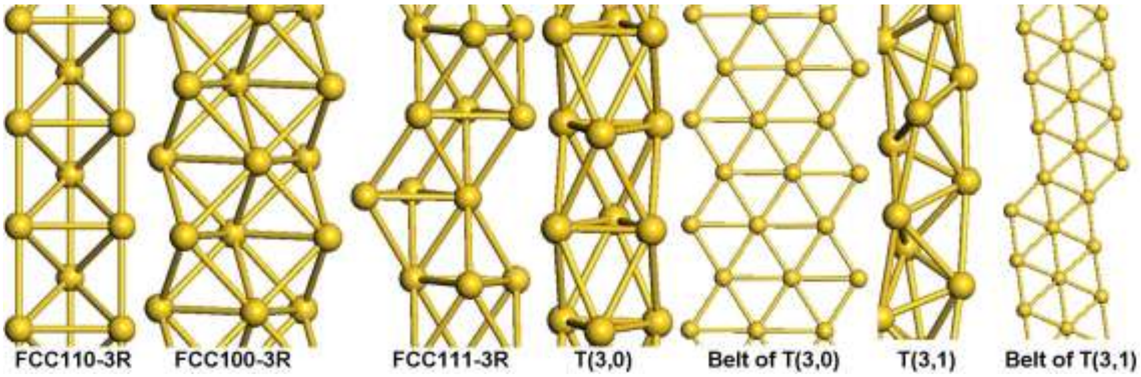

b. The side-view of 1D Au structures with four atomic rows:

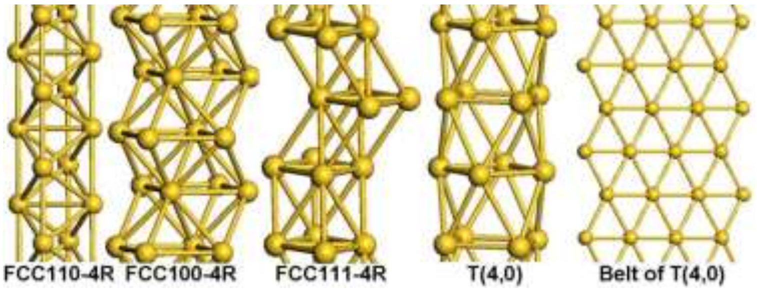

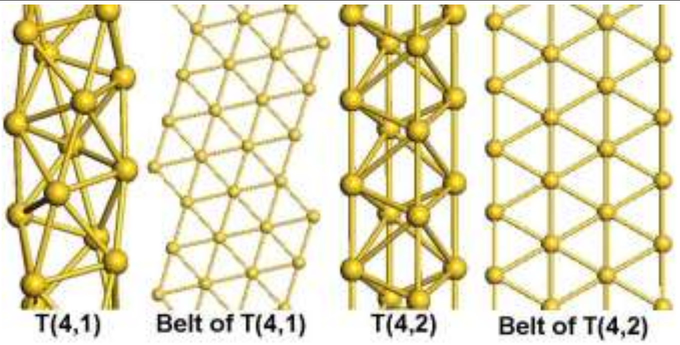

c. The side-view of 1D Au structures with five atomic rows:

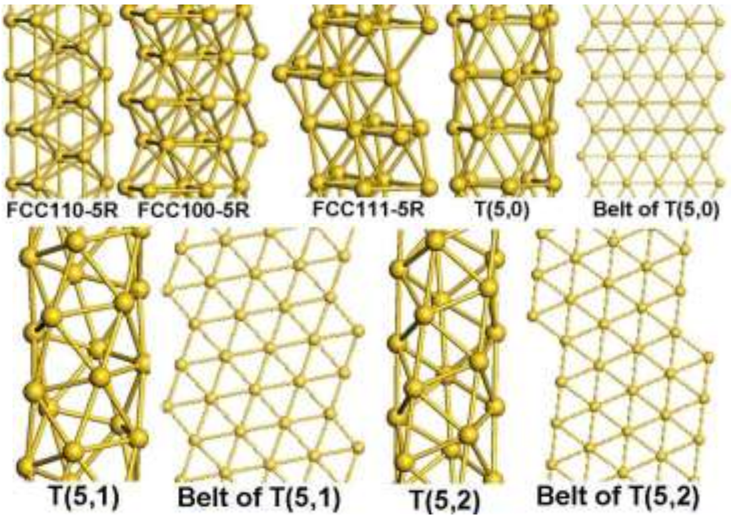

d. The side-view of 1D Au structures with six atomic rows:

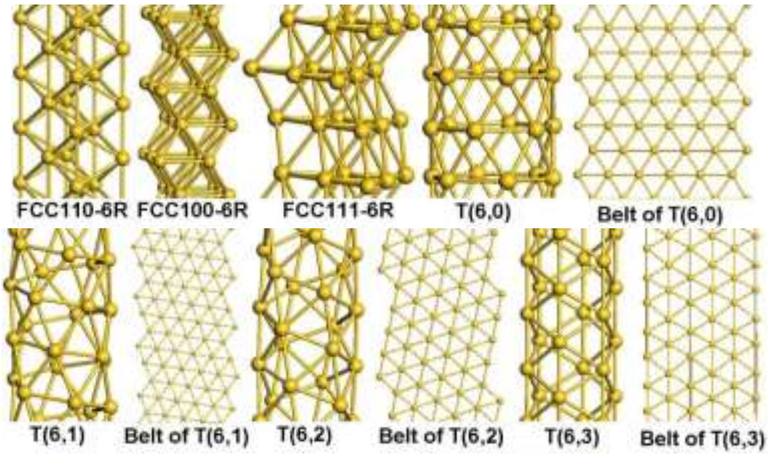

e. The cross-section of 1D Au structures with two shells:

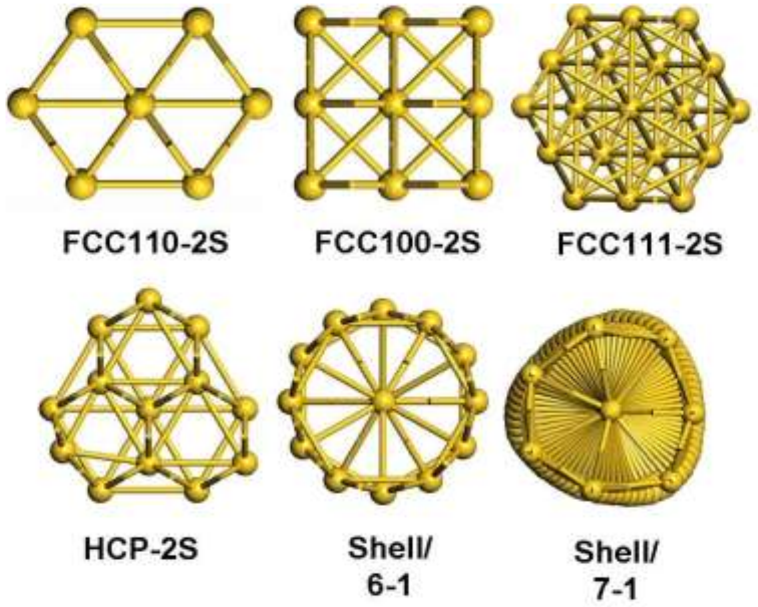

f. The cross-section of 1D Au structures with three shells:

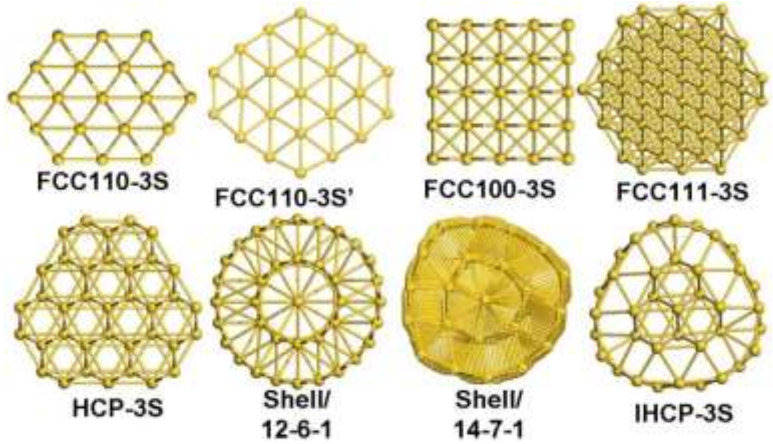

g. The cross-section of 1D Au structures with four shells:

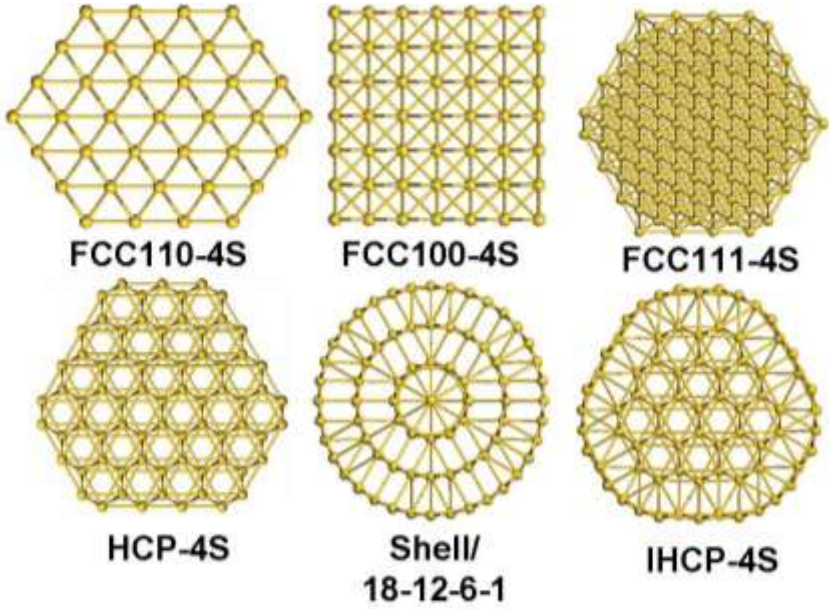

Figure S2. The radius and calculated E_c of Au cage, tube, and L_1.

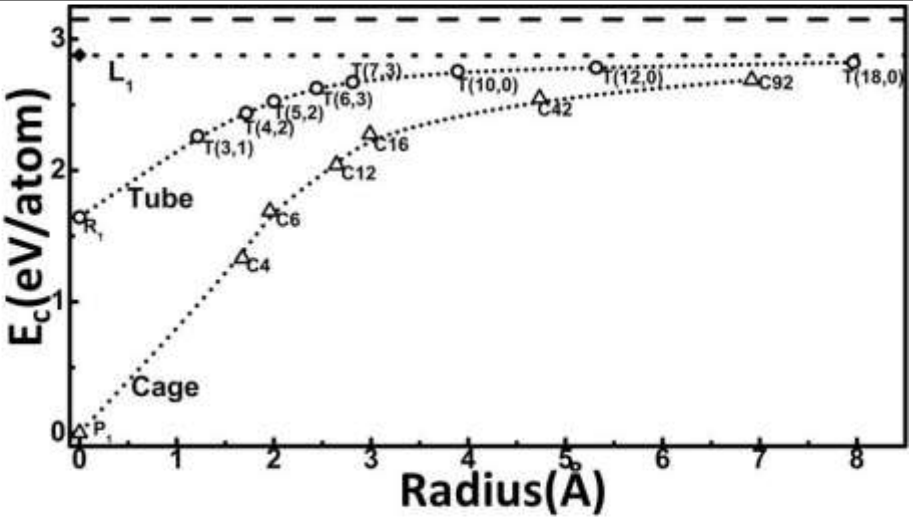

Figure S3. PDOS of Ag, Pt, Ir, Pd, Rh, Cu, Ni and Co structures.

3.1 PDOS of L_1 and FCC bulk of Ag

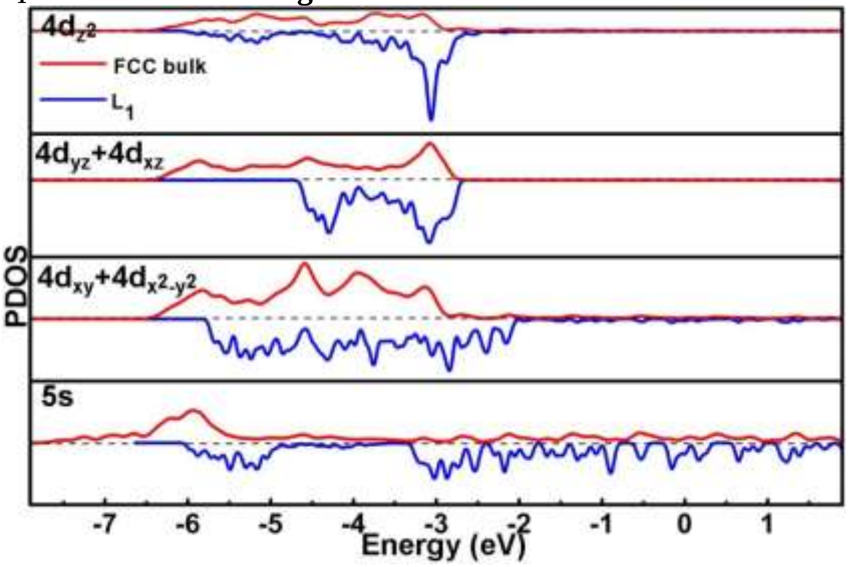

3.2 PDOS of L_1 and FCC bulk of Pt

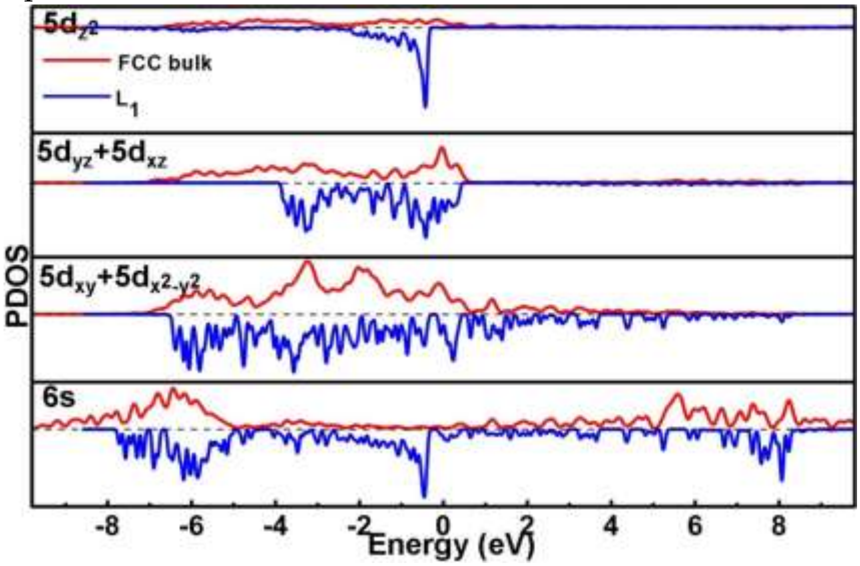

3.3 PDOS of L_1 and FCC bulk of Ir

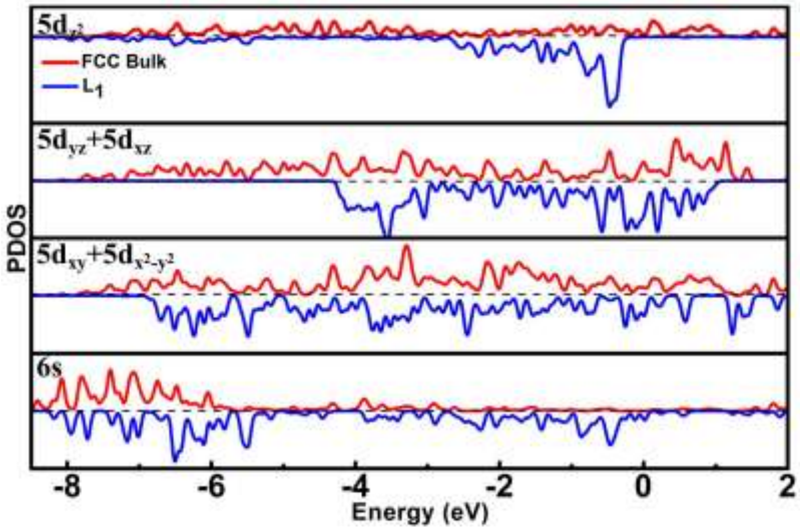

3.4 PDOS of L_1 and FCC bulk of Pd

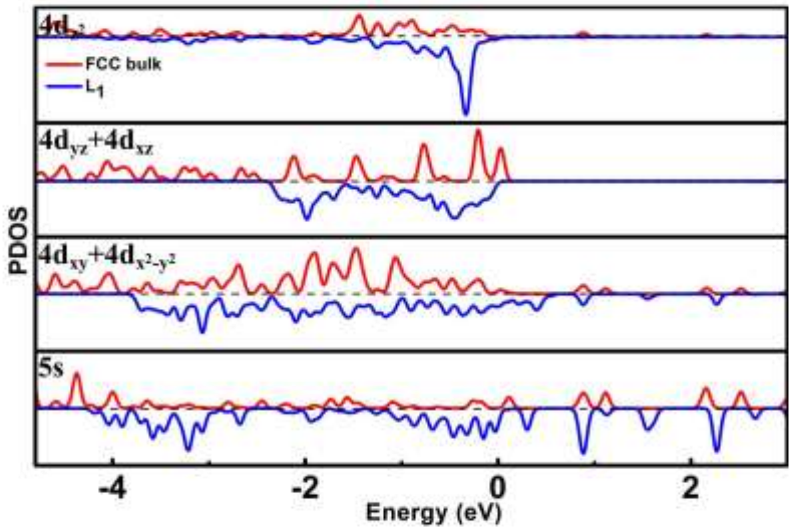

3.5 PDOS of L_1 and FCC bulk of Rh

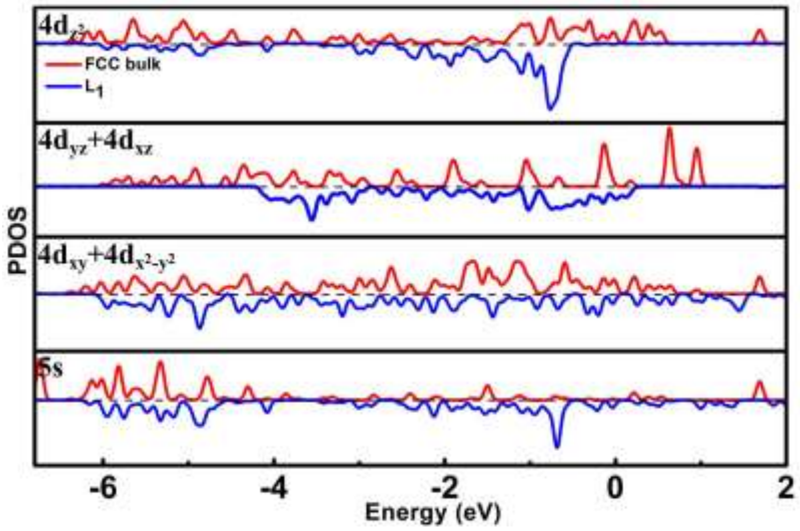

3.6 PDOS of L_1 and FCC bulk of Cu

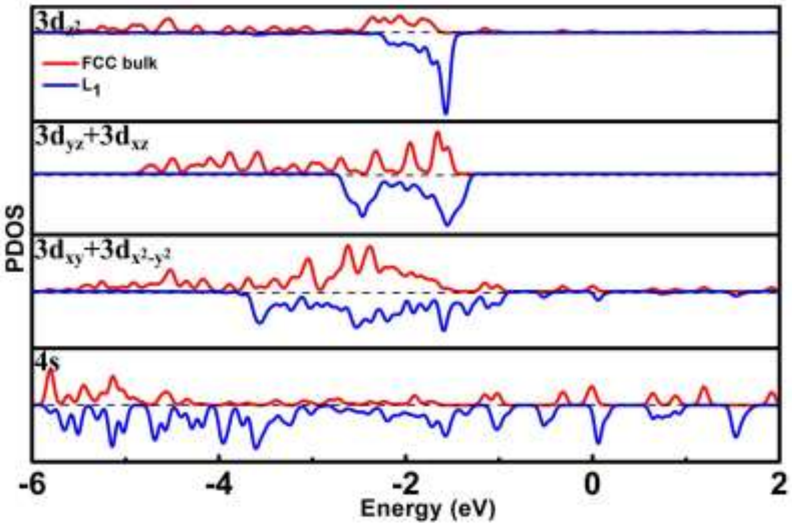

3.7 PDOS of L_1 and FCC bulk of Ni

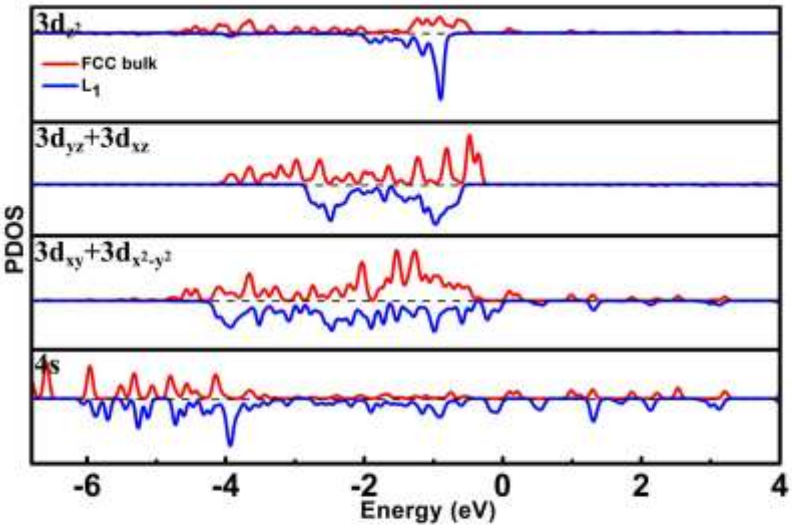

3.8 PDOS of L_1 and FCC bulk of Co

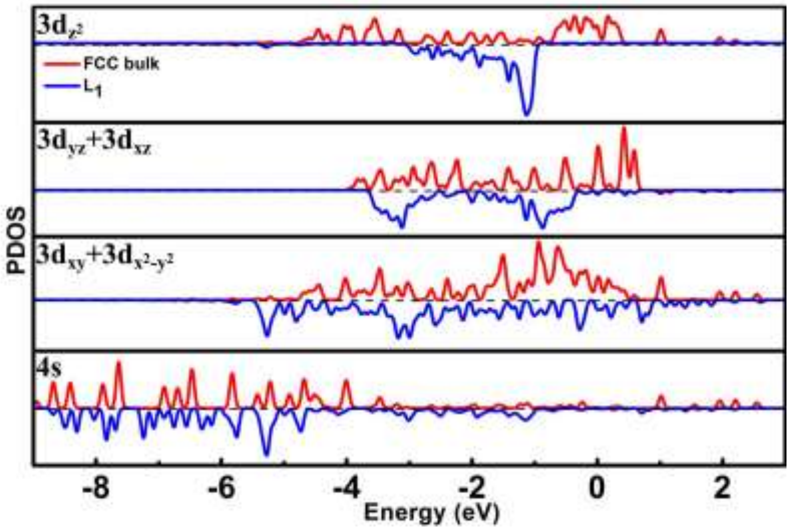

Figure S4. The comparison of calculated R_C with and without (in brackets) the consideration of scalar-relativistic effect. Here, the spin polarized calculations were performed by using $Dmol^3$ package [8]. GGA-PW91 functional and the double numerical basis set DNP were chosen [2, 9]. The all-electron core treatments were employed.

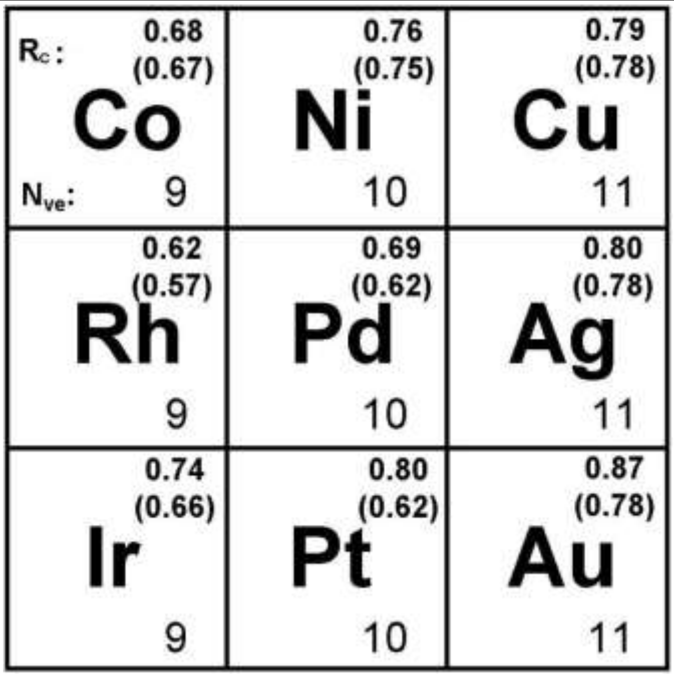

Figure S5. The calculated bond lengths and their variations (ΔL) caused by the scalar-relativistic effect of M-M in FCC bulk and L_1.

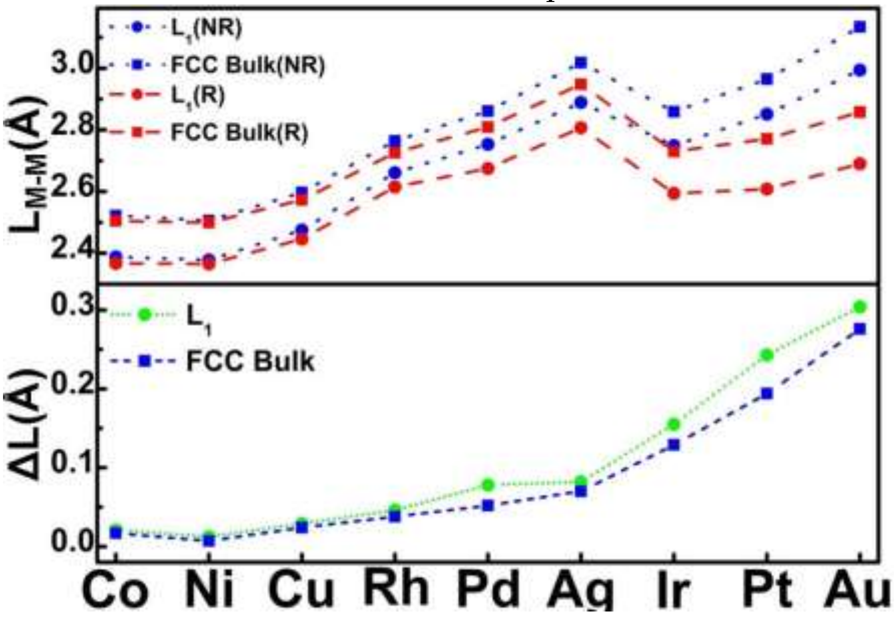

Figure S6. Structural reconstruction of FCC100 nanowires.

6.1 FCC100-3R

Atoms A, B, C, D of FCC100-3R are in (100) facet initially, while A and C move outward along the arrow to form bonds and eventually evolve to a FCC(111)-like surface. As a result, the original close-packed wire transforms to a hollow tube T(6,3).

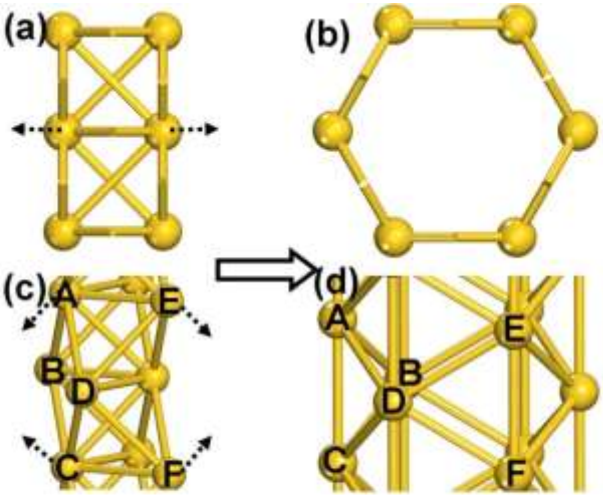

6.2. FCC100-4S

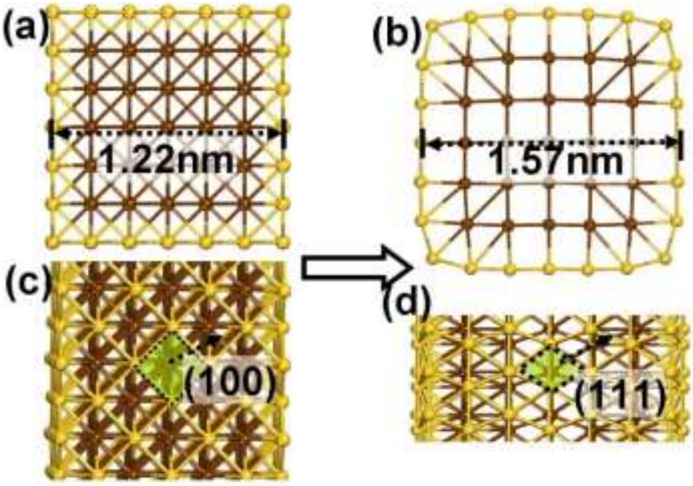

Figure S7. E_c of Au, Ag, and Cu nanowires

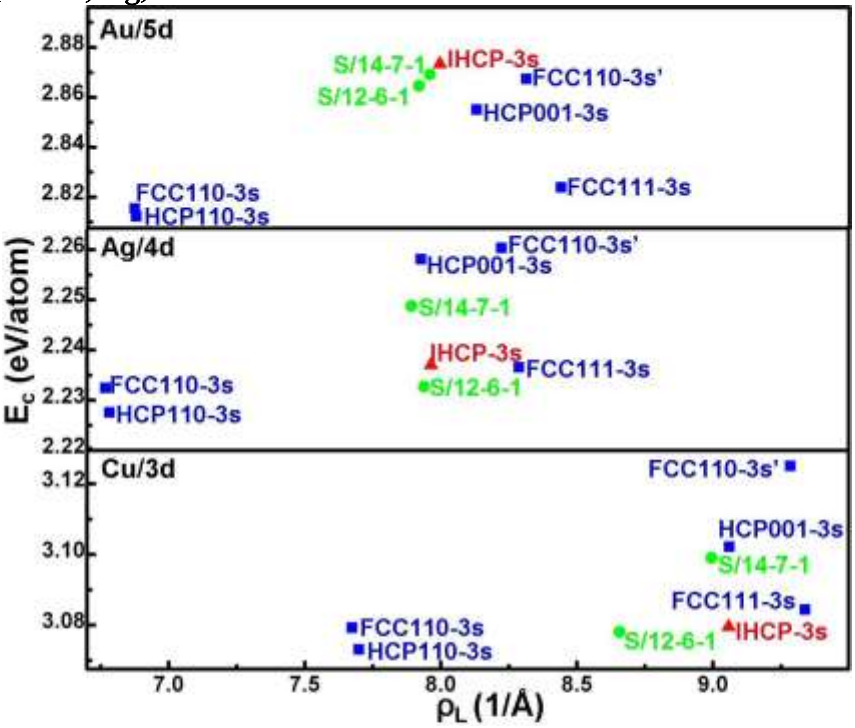

Figure S8. Adsorptions of O on (a) IHCP-3s, (b) Au_{38}, (c) FCC110-3s', and (d) Au(111) surfaces.

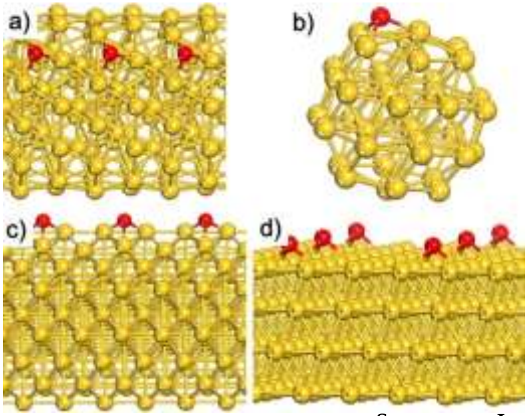

Figure S9. PDOS of the surface and inner atoms (Au^S and Au^I) of IHCP-3s.

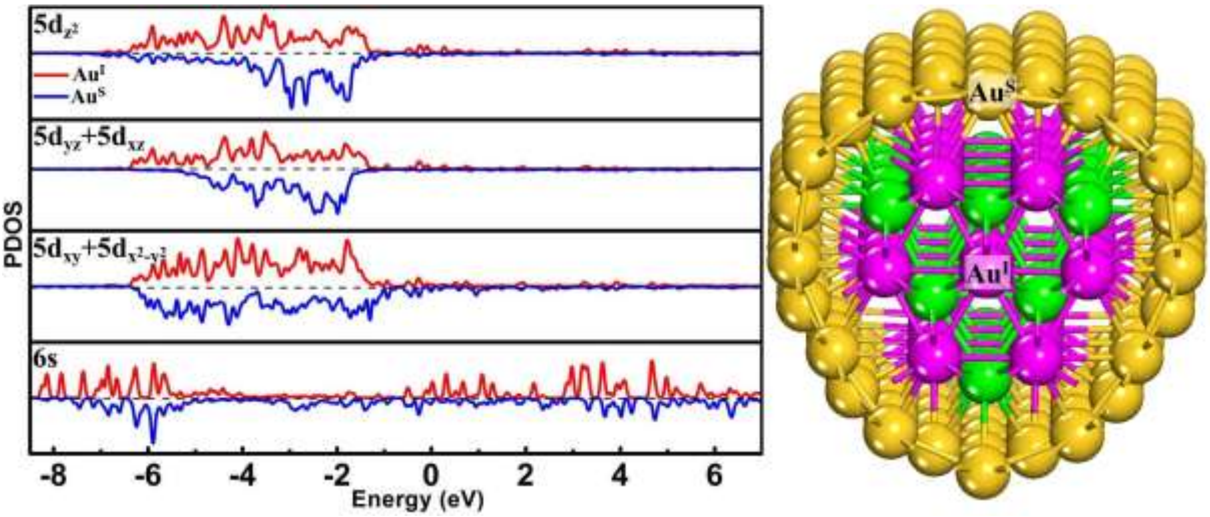

Table S1. Radius (R), E_c and O adsorption energies ($E_{O\text{-}ad}$) of different structures.

	R(Å)	E_c(eV/atom)	$E_{O\text{-}ad}$(eV)
L_1	0.00	2.88	2.63
T(6,0)	2.81	2.68	2.89
FCC100-2S	2.19	2.71	2.95
IHCP-3S	5.56	2.87	3.08
FCC bulk	∞	3.15, 3.19[1]	
Au(111)			3.10, 3.08[2]
Shell/6-1	2.80	2.67	3.03
T(5,2)	2.12	2.53	3.16, 3.20[3]
FCC110-2S	2.63	2.57	3.24
FCC111-2S	2.63	2.58	3.41

[1] T. Nautiyal, S. J. Youn, and K. S. Kim, *Phys. Rev. B* **2003,** 68, 033407

[2] T. A. Baker, C. M. Friend, and E. Kaxiras, *J. Phys. Chem. C* **2009,** 113, 3232.

[3] W. An, Y. Pei, and X. C. Zeng, *Nano Lett.* **2008,** 8, 195.
